# Different responses to treatment across classified diseases and severities in Japanese patients with microscopic polyangiitis and granulomatosis with polyangiitis: a nationwide prospective inception cohort study

**DOI:** 10.1186/s13075-015-0815-y

**Published:** 2015-11-02

**Authors:** Ken-ei Sada, Masahiro Yamamura, Masayoshi Harigai, Takao Fujii, Yoshinari Takasaki, Koichi Amano, Shouichi Fujimoto, Eri Muso, Yohko Murakawa, Yoshihiro Arimura, Hirofumi Makino, for the Research Committee on Intractable Vasculitides, the Ministry of Health, Labour and Welfare of Japan

**Affiliations:** Department of Medicine and Clinical Science, Okayama University Graduate School of Medicine, Dentistry and Pharmaceutical Sciences, 2-5-1 Shikata-cho, Kita-ku, Okayama 700-8558 Japan; Center for Rheumatology, Okayama Saiseikai General Hospital, 1-17-18 Ifuku-cho, Kita-ku, Okayama 700-8511 Japan; Department of Pharmacovigilance, Graduate School of Medical and Dental Sciences, Tokyo Medical and Dental University, 1-5-45 Yushima, Bunkyo-ku, Tokyo 113-8519 Japan; Department of Medicine and Rheumatology, Graduate School of Medical and Dental Sciences, Tokyo Medical and Dental University, 1-5-45 Yushima, Bunkyo-ku, Tokyo 113-8519 Japan; Department of the Control for Rheumatic Diseases, Graduate School of Medicine, Kyoto University, 54 Shogoin-Kawahara-cho, Sakyo-ku, Kyoto 606-8507 Japan; Department of Internal Medicine and Rheumatology, Juntendo University School of Medicine, 2-1-1 Hongo, Bunkyo-ku, Tokyo 113-8431 Japan; Department of Rheumatology and Clinical Immunology, Saitama Medical Center, Saitama Medical University, 1981 Kamoda, Saitama, 350-8550 Japan; Department of Hemovascular Medicine and Artificial Organs, Faculty of Medicine, University of Miyazaki, 5200 Kiyotakecho Kihara, Miyazaki, 889-1692 Japan; Center for Nephrology and Urology, Division of Nephrology and Dialysis, Kitano Hospital, Tazuke Kofukai Medical Research Institute, 2-4-20 Ohgimachi, Osaka, 530-8480 Japan; Department of Rheumatology, Shimane University Faculty of Medicine, 89-1 Enya-cho, Izumo, 693-8501 Japan; Nephrology and Rheumatology, First Department of Internal Medicine, Kyorin University School of Medicine, 6-20-2 Shinkawa, Mitaka, Tokyo 181-8611 Japan; Okayama University Hospital, 2-5-1 Shikata-cho, Kita-ku, Okayama 700-8558 Japan

**Keywords:** Antineutrophil cytoplasmic antibody-associated vasculitis, Cyclophosphamide, Glucocorticoid, Granulomatosis with polyangiitis, Inception cohort, Microscopic polyangiitis, Prospective cohort

## Abstract

**Introduction:**

This study aims to elucidate the prognosis and the effectiveness of current treatments for Japanese patients with microscopic polyangiitis (MPA) and granulomatosis with polyangiitis (GPA).

**Methods:**

Patients with newly diagnosed MPA and GPA were enrolled in a nationwide, prospective, inception cohort study from 22 tertiary Japanese institutions, and treatment patterns and responses were evaluated for 24 months. Primary outcome measures were rates of remission (Birmingham Vasculitis Activity Score, 0) and remission with low-dose glucocorticoids (GC) (prednisolone ≤ 10 mg) (GC remission).

**Results:**

Of 156 enrolled patients, 78 MPA patients and 33 GPA patients were included. Concomitant cyclophosphamide (CY) was used in 24 MPA (31 %) and 20 GPA (60 %) patients during the initial 3 weeks of treatment. After 6 months, remission was achieved in 66 MPA (85 %) and 29 GPA (87 %) patients, while GC remission was obtained in only 31 MPA (40 %) and 13 GPA (39 %) patients. During the 24-month period, 14 MPA patients and 2 GPA patients died; end stage renal disease (ESRD) was noted in 13 MPA patients but no GPA patients. Patients with severe disease, according to the European Vasculitis Study Group (EUVAS) classification, showed poorer ESRD-free and overall survival rates than those with generalized disease (p < 0.0001). There were no differences in relapse-free survival rates between GPA and MPA, among EUVAS-defined disease severity categories, and between anti-neutrophil cytoplasmic antibody subspecialties.

**Conclusions:**

The majority of Japanese patients with MPA and GPA received treatment with high-dose GC and limited CY use, and showed high remission and relapse-free survival rates but low GC remission rates in clinical practice.

**Trial registration:**

University Hospital Medical Information Network Clinical Trials Registry UMIN000001648. Registered 28 February 2009.

## Introduction

Antineutrophil cytoplasmic antibody (ANCA)-associated vasculitis (AAV) is characterized by blood vessel inflammation with few or no immune deposits and the presence of ANCA positivity. Recently, the 2012 revised International Chapel Hill Consensus Conference Nomenclature of Vasculitides classified AAV into four clinically relevant subsets: microscopic polyangiitis (MPA), granulomatosis with polyangiitis (GPA), eosinophilic granulomatosis with polyangiitis (EGPA), and renal-limited AAV (RLV), considered an organ-specific variant of MPA [1]. Watts et al. [2] proposed an epidemiological classification algorithm (the European Medicines Agency (EMEA) algorithm), which classifies primary systemic vasculitis into EGPA, GPA, MPA/RLV, and polyarteritis nodosa, with no overlaps between the categories. Significant differences in clinical characteristics and ANCA serology of AAV between European and Asian patients have been suggested [3]. A European epidemiologic study showed a dominance of GPA over MPA [4], while our prospective study [5] and two retrospective studies from China [6] and Japan [3] that applied the EMEA algorithm found perinuclear/myeloperoxidase (MPO)-ANCA-positive MPA to be the most common form of AAV in Asia.

The guidelines of the British Society of Rheumatology (BSR) and the European League Against Rheumatism (EULAR) recommended assessment for treatment with high-dose glucocorticoids (GC) and concomitant cyclophosphamide (CY) or rituximab as the first-line option for all patients with newly diagnosed AAV, in principle [7, 8]. These recommendations are based on several randomized controlled trials (RCTs) enrolling more patients with GPA than with MPA [9–11]. Since patients with MPA were older and tended to exhibit higher levels of creatinine than those with GPA in our cohort of Japanese patients [5], these recommendations should be applied to Asian patients with caution in terms of safety.

Recently, Ozaki et al. [12] have reported the treatment effectiveness stratified by disease severity for Japanese patients with MPO-ANCA-positive MPA in a prospective observational study. However, differences in treatment effectiveness between MPA and GPA patients and across the spectrum of disease severity remain to be clarified.

We have previously reported the demographic and baseline clinical characteristics of Japanese patients with AAV who were enrolled in a nationwide, prospective, inception cohort study of Remission Induction Therapy in Japanese patients with ANCA-associated Vasculitides (RemIT-JAV). In the present study, we aimed to elucidate and compare treatment and its effectiveness in terms of remission, survival, and relapse rates in clinical practice in Japanese patients with MPA and GPA using the RemIT-JAV cohort database.

## Methods

### Settings and patient population

#### Database

Details regarding the RemIT-JAV study protocol have been reported previously [5]. Twenty-two tertiary care institutions participated in this study and enrolled 156 consecutive patients with newly diagnosed AAV from April 2009 to December 2010. The criteria for enrolment included a diagnosis of AAV made by the site investigators that fulfilled the criteria for primary systemic vasculitis proposed by the EMEA algorithm: symptoms and signs characteristic of or compatible with a diagnosis of AAV or polyarteritis nodosa; at least one of histological proof of vasculitis, positive serology for ANCA, specific investigations strongly suggestive of vasculitis and/or granuloma, or eosinophilia; and no other diagnosis to account for symptoms/signs [2]. “Specific investigations” included neurophysiological tests for mononeuritis multiplex, conventional or magnetic resonance angiography, and thoracic or neck magnetic resonance imaging/computed tomography imaging, depending on the signs and symptoms of the patients. The site investigators excluded patients with malignancy, infection, drug-induced vasculitis, secondary vasculitis, other types of vasculitides, vasculitis mimics, sarcoidosis, and other nonvasculitic granulomatous disease. Of the 156 patients enrolled, 78, 33, and 14 were classified as MPA, GPA, and EGPA, respectively. The present study included all patients with MPA or GPA from the RemIT-JAV study, but not those with EGPA. The RemIT-JAV study was conducted according to the Declaration of Helsinki and the ethical guidelines for epidemiologic research in Japan. Written informed consent was obtained from each participant, and the study protocol was approved by the ethics committees at each participating institution (refer to Acknowledgements). The RemIT-JAV study was registered to the University Hospital Medical Information Network Clinical Trials Registry (UMIN000001648).

#### Data collection

Each patient’s baseline data included demographic information, comorbidities, laboratory data, Birmingham Vasculitis Activity Score (BVAS), imaging data, and respiratory function data. Patients were evaluated at 3, 6, 12, 18, and 24 months after diagnosis and at relapse, and the following data were collected: vital status, BVAS, laboratory data, treatments, and adverse events. Organ involvement was defined according to BVAS system. Disease severity was determined by the categorization system developed by the European Vasculitis Study Group (EUVAS) [13]. The Vascular Damage Index (VDI) was also collected at 6, 12, and 24 months. Chest radiography, thoracic computed tomography, arterial blood gas analysis, and respiratory function data were collected at 12 and 24 months in patients with pulmonary involvement. Because the RemIT-JAV study was an observational study, a treatment protocol was not provided. Selection and dosage of immunosuppressants, dosage of GC, and concomitant usage of plasmapheresis were determined at the discretion of the attending physicians. Observation was completed in March 2013, and the baseline characteristics have been described previously [5].

Site investigators filled out the electronic case report forms for each patient and submitted them to the RemIT-JAV data center at the Department of Medicine and Clinical Science, Okayama University Graduate School of Medicine, Dentistry and Pharmaceutical Sciences, Okayama, Japan.

### Primary and secondary outcomes

The primary effectiveness outcome of the present study was the remission rate. We analyzed two types of remission in this study. The first type of remission was determined systematically by a BVAS of 0 on two occasions at least 1 month apart according to EULAR recommendations for conducting clinical studies and/or clinical trials in systemic vasculitis [13]. The second type of remission was defined as BVAS of 0 plus a daily prednisolone (PSL) dose of ≤10 mg (designated as “GC remission” hereafter). GCs other than PSL were converted to an equivalent dose of PSL.

Secondary effectiveness outcomes included cumulative overall end-stage renal disease (ESRD)-free and relapse-free survival rates. ESRD was defined as dependence on dialysis or an irreversible increase in serum creatinine level of >5.6 mg/dl (500 μmol/l). Based on the aforementioned EULAR recommendations, relapse was defined as the reoccurrence or new onset of disease activity attributable to active inflammation. Major relapse was defined as a relapse with organ-threatening or life-threatening disease activity, and other relapses were classified as minor [13].

The safety outcome was the type and incidence of serious infections (SIs). Our definition of SIs was based on an International Conference on Harmonisation report [14]. Bacterial infections that required intravenous antibiotic administration and opportunistic infections were regarded as SIs. The diagnosis of infection was based on the attending physicians’ clinical diagnosis, using a comprehensive evaluation of physical findings, laboratory data, and imaging data.

### Statistical analysis

We used baseline and follow-up data at 3, 6, 12, 18, and 24 months from the patients with MPA and GPA enrolled in this study. The cumulative remission, overall survival, ESRD-free survival, and relapse-free survival rates were analyzed using the Kaplan–Meier method and the log-rank test. *p* <0.05 was considered significant for statistical analyses between two categories. When comparing three or four categories, statistical significance was determined by *p* <0.05/3 or *p* <0.05/4 by Bonferroni correction to avoid multiplicity. When no patients in a group achieved an endpoint, the group was excluded from the long-rank test. As such, the patients with limited type disease, as defined by EUVAS categorization [13], were excluded from the analysis of survival and ESRD-free survival, and patients with early systemic type disease were excluded from the analysis of ESRD-free survival.

All statistical analyses were performed by a biostatistician using the Statistical Package of JMP for Windows software, version 8.0.2 (SAS Institute Inc., Cary, NC, USA).

## Results

### Patient characteristics

**Fig. 1:**
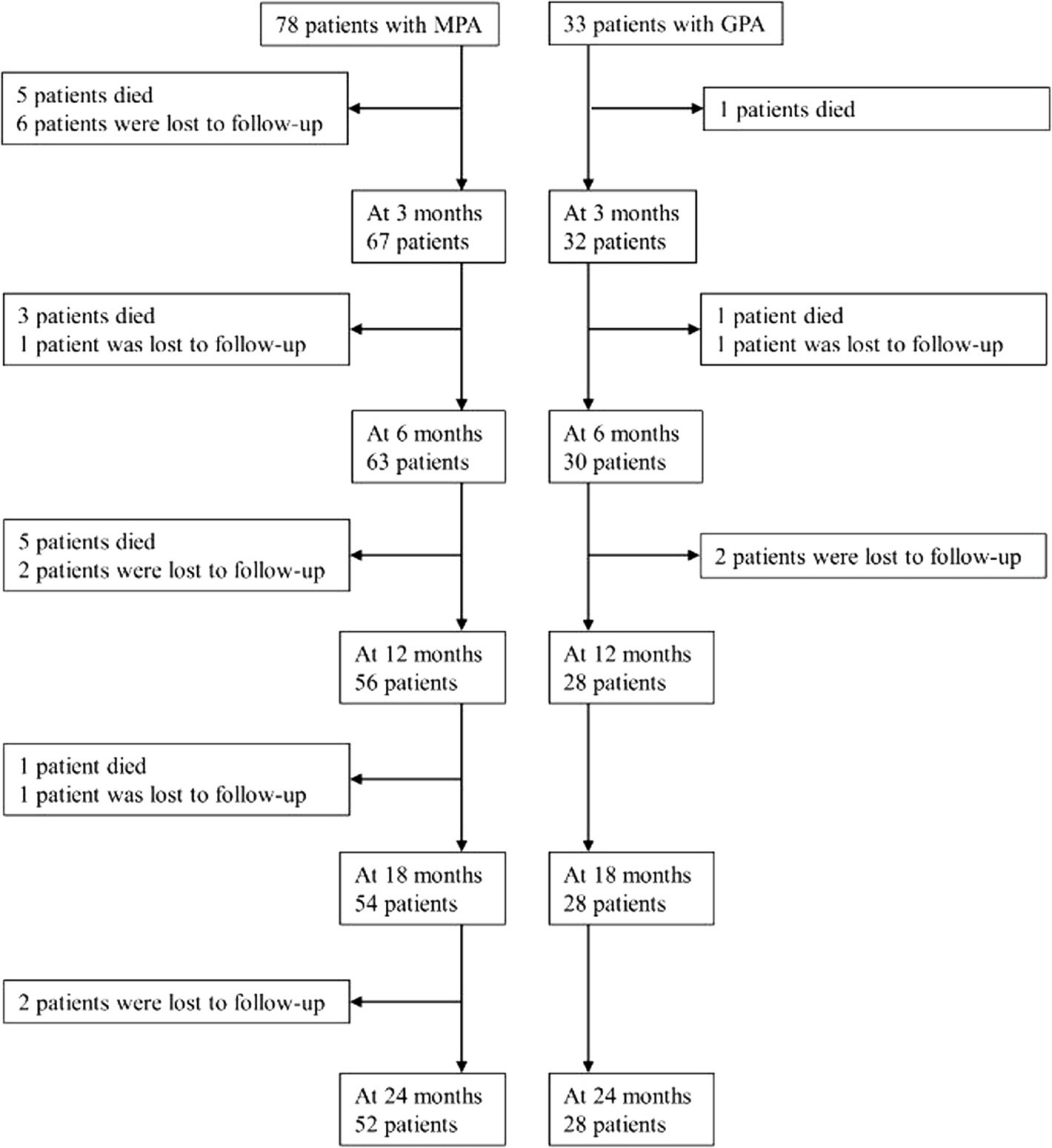
Flow chart of the enrolled patients. *GPA* granulomatosis with polyangiitis, *MPA* microscopic polyangiitis

Of the 156 patients with AAV enrolled in the RemIT-JAV study, 78 patients with MPA/RLV and 33 patients with GPA were included in the present study. The mean (median) observation periods were 559 (730) days in MPA patients and 653 (730) days in GPA patients, respectively. At 24 months, 52 patients with MPA and 28 patients with GPA remained in the cohort (Fig. 1). Selected patient characteristics and treatment of these patients are summarized in Table 1; details regarding patient characteristics have been described in our previous report [5].

**Table 1:** Patient characteristics and treatments

	MPA	GPA
	(*n* = 78)	(*n* = 33)
Age (years), mean ± SD	71 ± 10	64 ± 13
Sex (male:female)	35:43	12:21
Disease severity, *n*		
Limited	0	4
Early systemic	15	5
General	47	18
Severe	16	6
MPO-ANCA, *n* (%)	76 (97)	18 (55)
PR3-ANCA, *n* (%)	2 (3)	15 (46)
Serum creatinine (mg/dl), mean ± SD	2.46 ± 2.18	1.51 ± 1.32
BVAS, mean ± SD	18 ± 7	20 ± 7
Initial treatments (within 3 weeks)		
Maximum daily dose of PSL (mg/day), mean ± SD	41 ± 15	40 ± 15
mPSL pulse use, *n* (%)	34 (44)	13 (39)
Cyclophosphamide, *n* (%)	24 (31)	20 (60)
Oral:intravenous	3:21	7:13
Cumulative dose for 6 months (g), mean ± SD	2.6 ± 2.8	4.3 ± 3.6
Methotrexate, *n* (%)	0 (0)	2 (6)
Azathioprine, *n* (%)	0 (0)	0 (0)
Treatments at 6 months		
	*n* = 62	*n* = 30
Minimum daily dose of PSL (mg/day), mean ± SD	12 ± 5	13 ± 6
Cyclophosphamide, *n*	20	16
Methotrexate, *n*	0	3
Azathioprine, *n*	10	8
Treatments at 12 months		
	*n* = 56	*n* = 28
Minimum daily dose of PSL (mg/day), mean ± SD	9 ± 5	9 ± 6
Cyclophosphamide, *n*	9	7
Methotrexate, *n*	0	4
Azathioprine, *n*	11	11
Treatments at 24 months		
	*n* = 52	*n* = 28
Minimum daily dose of PSL (mg/day), mean ± SD	7 ± 5	6 ± 3
Cyclophosphamide, *n*	4	5
Methotrexate, *n*	0	4
Azathioprine, *n*	10	5

*ANCA* antineutrophil cytoplasmic antibody, *BVAS* Birmingham Vasculitis Activity Score, *GPA* granulomatosis with polyangiitis, *MPA* microscopic polyangiitis, *MPO* myeloperoxidase, *mPSL* methylprednisolone, *PR3* proteinase 3, *PSL* prednisolone, *SD* standard deviation

### Treatment patterns

Changes in treatment over time in patients with MPA and GPA are presented in Table 1 as observed data. The mean initial daily PSL dose was 41 mg in MPA patients and 40 mg in GPA patients. Concomitant CY was used during the initial 3 weeks of remission induction therapy in 24 of 78 (31 %) MPA patients and 20 of 33 (60 %) GPA patients. One GPA patient and three MPA patients were treated with plasma exchange. Hemodialysis was implemented at any time point during the initial 3 months in 13 of 78 (14 %) MPA patients and 2 of 33 (6 %) GPA patients. At 3 months, the daily PSL dose was tapered down to ≤15 mg in 29 of 67 (43 %) MPA patients and 10 of 32 (31 %) GPA patients.

At 6 months, the mean daily PSL dose was 12 mg in MPA patients and 13 mg in GPA patients, and 10 of 62 MPA patients (16 %) and 8 of 30 GPA patients (27 %) were receiving concomitant azathioprine. Data regarding treatment were not obtained in one patient with MPA. The mean daily PSL dose decreased gradually thereafter in both groups.

### Remission

**Fig. 2:**
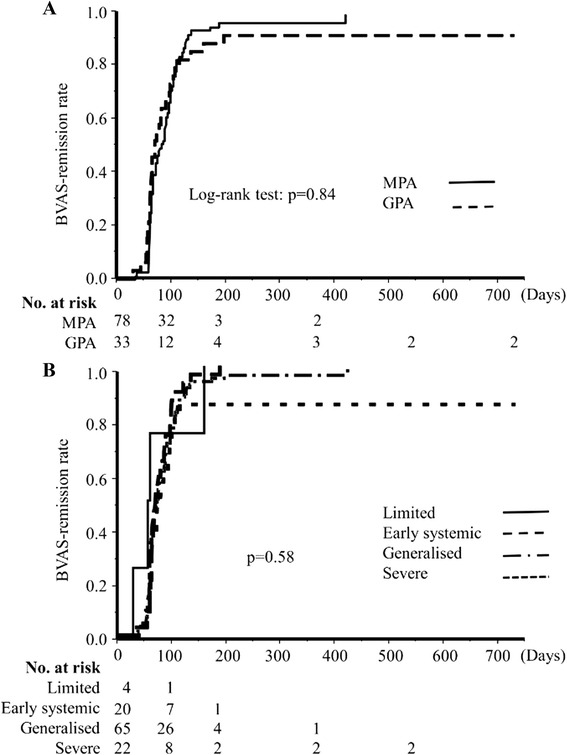
Cumulative remission rates in MPA and GPA patients and among the four EUVAS-defined disease severity types. **a** Remission rates in MPA and GPA patients. **b** Remission rates among patients with the four types of EUVAS disease severity. *BVAS* Birmingham Vasculitis Activity Score, *GPA* granulomatosis with polyangiitis, *MPA* microscopic polyangiitis

No statistically significant differences in remission rates were found between MPA and GPA patients (Fig. 2a). By 6 months, 66 of 78 MPA patients (85 %) and 29 of 33 GPA patients (87 %) achieved remission, as defined by BVAS of 0 on two occasions at least 1 month apart. However, only 31 of 78 MPA patients (40 %) and 13 of 33 GPA patients (39 %) satisfied the definition of GC remission; that is, BVAS of 0 remission plus a daily PSL dose of ≤10 mg.

When both patients with MPA and those with GPA were classified into four different types (i.e. limited, early systemic, generalized, and severe) according to the EUVAS-defined disease severity, there were no significant differences in remission rates across the spectrum of disease severity (Fig. 2b). Remission and GC remission were obtained by 6 months in four (100 %) and three (75 %) of four patients with the limited type, in 18 (90 %) and 10 (50 %) of 20 patients with the early systemic type, in 58 (89 %) and 24 (37 %) of 65 patients with the generalized type, and in 15 (68 %) and 7 (32 %) of 22 patients with the severe type, respectively.

No significant differences in remission and GC remission rates at 6 months were noted between MPO-ANCA-positive and peroxidase-3 (PR3)-ANCA-positive patients, or between patients with or without interstitial lung disease (ILD).

### Overall survival

Sixteen deaths were reported during the observation period (14 MPA patients and two GPA patients). Causes of death reported by site investigators were vasculitis itself (five patients), infection (six patients), and other or unknown (five patients: completely unknown in two patients, cardiac failure in one patient, intracerebral hemorrhage in one patient, and hyperkalemia in one patient). Of the five cases of death caused by vasculitis itself, all patients were treated with GC without concomitant CY and two patients were treated with plasma exchange. Renal manifestations developed in all five cases and ESRD developed in two of five cases. Lung manifestations developed in four of five patients including pulmonary hemorrhage and ILD in one case, ILD in one case, pleuritis in one case, and pulmonary infiltration in one case.

**Fig. 3:**
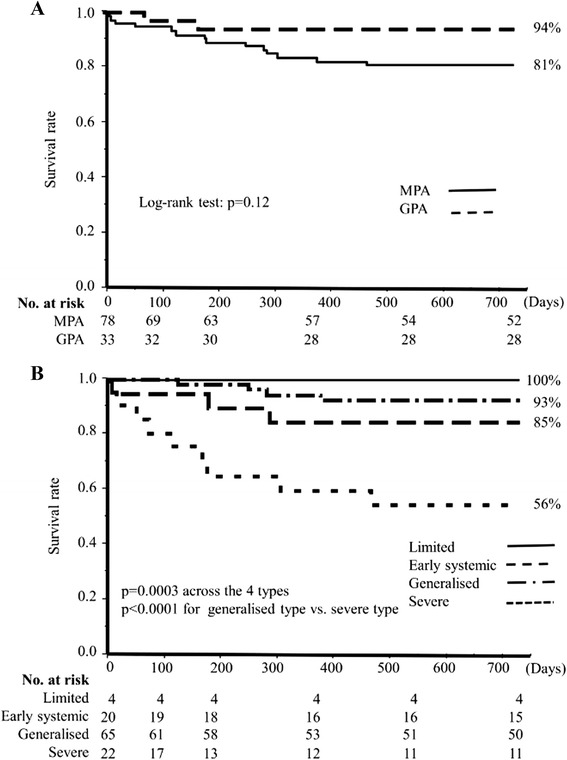
Cumulative overall survival rates in MPA and GPA patients and among the four EUVAS-defined disease severity types. **a** Survival rates in MPA and GPA patients. **b** Survival rates among patients with the four types of EUVAS disease severity. Cumulative survival rates at 24 months are shown at the end of the survival curve for each group. *GPA* granulomatosis with polyangiitis, *MPA* microscopic polyangiitis

In terms of overall survival rates, MPA patients tended to have a worse prognosis than GPA patients, although the difference did not reach statistical significance (Fig. 3a; *p* = 0.12). In the spectrum of EUVAS disease severity, there were significant differences in overall survival rates among the four different types (Fig. 3b; *p* = 0.0003). Patients with severe disease, showing the lowest survival rate of 56 % at 24 months, had a markedly poorer prognosis, even compared with those with generalized type disease (*p* <0.0001).

On the other hand, significant differences in survival rates were undetectable between MPO-ANCA-positive and PR3-ANCA-positive patients (82 % versus 92 % at month 24) or between patients with and without ILD (87 % versus 83 % at month 24).

### ESRD-free survival

**Fig. 4:**
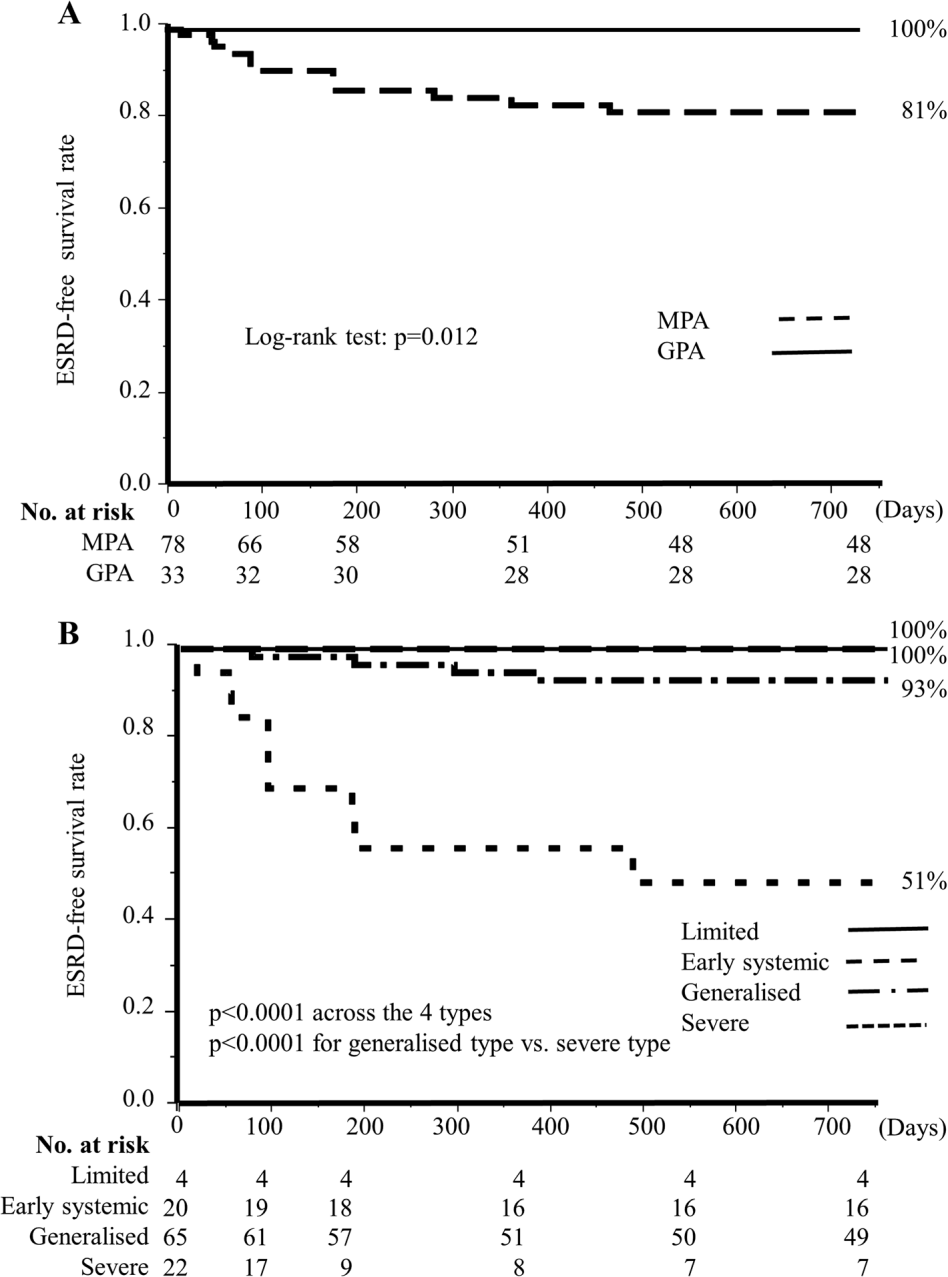
Cumulative ESRD-free survival in MPA and GPA patients and among the four EUVAS-defined disease severity types. **a** ESRD-free survival rates in MPA and GPA patients. **b** ESRD-free survival rates among patients with the four types of EUVAS disease severity. Cumulative ESRD-free survival rates at 24 months are shown at the end of the survival curve for each group. *ESRD* end-stage renal disease, *GPA* granulomatosis with polyangiitis, *MPA* microscopic polyangiitis

Thirteen cases of ESRD developed during the observation period in MPA patients, but none occurred in GPA patients. Hence, the ESRD-free survival rate of MPA patients was significantly lower than that of GPA patients, decreasing to 81 % at 24 months (Fig. 4a; *p* = 0.012). In the spectrum of EUVAS disease severity, there were significant differences in ESRD-free survival rates (Fig. 4b; *p* <0.0001). Patients with severe type disease showed the lowest ESRD-free survival rate of 51 % at 24 months, which was much lower than that of patients with generalized type disease (*p* <0.0001).

MPO-ANCA-positive patients tended to have lower ESRD-free survival rates than PR3-ANCA-positive patients (84 % versus 100 % at 24 months), although this difference was not statistically significant. No significant difference in ESRD-free survival rates was detectable between patients with and without ILD (90 % versus 85 % at 24 months).

### Relapse

**Fig. 5:**
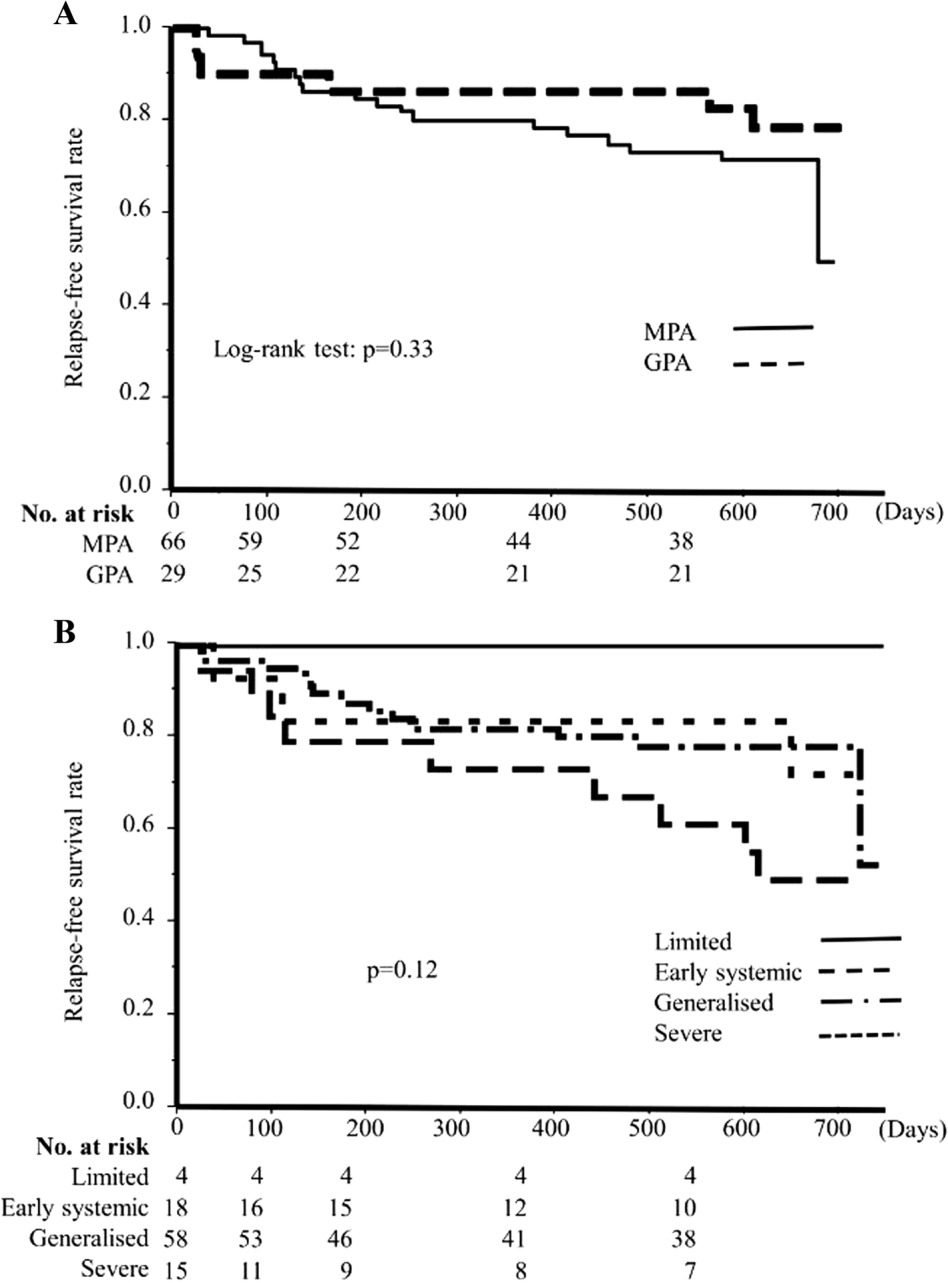
Relapse-free survival in MPA and GPA patients and among the four EUVAS-defined disease severity types. **a** Relapse-free survival rates in MPA and GPA patients. **b** Relapse-free survival rates among patients with the four types of EUVAS disease severity. *GPA* granulomatosis with polyangiitis, *MPA* microscopic polyangiitis

Of the 98 patients who achieved remission by 6 months, 23 relapsed during the total observation period, including 18 MPA and five GPA patients, with a mean duration for the remission period of 566 days. Of the 23 relapses, 11 were major relapses and 12 were minor. There were no significant differences in relapse rates between MPA and GPA patients (Fig. 5a; 29 % versus 15 % at month 18) or among patients with the four different types of EUVAS disease severity (Fig. 5b) (0 %, 42 %, 21 %, and 28 % at month 18 for limited, early systemic, generalized, and severe types, respectively). In addition, there were no significant differences in relapse rates between MPO-ANCA-positive and PR3-ANCA-positive patients (25 % versus 21 % at month 18) or between patients with or without ILD (34 % versus 18 % at month 18).

### SI and VDI

During the 2-year follow-up period, 76 SIs were reported in 46 patients (41.4 %). The number of SIs decreased over time, from 43 in 27 patients in the first 6 months to 20 in 18 patients in the second 6 months, nine in seven patients in the third 6 months, and four in four patients in the last 6 months of follow-up. The most frequent site of SI was the respiratory system (41 events), followed by skin and subcutaneous tissue (12 events). Frequently reported types of opportunistic infections were deep mycoses (14 events), cytomegalovirus infection (10 events), and herpes zoster (nine events).

The mean (standard deviation) total VDI score of MPA patients and GPA patients were 1.95 (1.55) and 1.64 (1.57) at 12 months and 2.13 (1.56) and 2.11 (1.79) at 24 months, respectively.

## Discussion

This is the first prospective cohort study comparing the responses of current immunosuppressive therapy in Japanese patients between the two major AAV diseases, MPA and GPA, in clinical practice. In our cohort, the patients had been enrolled from 22 tertiary care institutions in Japan. The majority was positive for MPO-ANCA and diagnosed with MPA, which is in contrast with AAV patients in western countries who are characterized by a predominance of PR3-ANCA and GPA. In addition, although the EULAR recommendations and the BSR guidelines recommend the concomitant use of CY as induction therapy for severe or generalized AAV [7, 8], concomitant CY was used in only 31 % of MPA patients and 60 % of GPA patients during remission induction, and these rates were apparently lower than those reported in western observational studies (76–82 %) [15–17]. In previous studies, CY was not frequently used in Japan for patients with MPA/RLV, because these patients often present with progressive renal impairment at advanced ages and are thereby considered at high risk for severe infections [15, 18].

In this study, we found that remission rates (BVAS of 0 on two occasions at least 1 month apart) at 6 months were 85 % for MPA and 87 % for GPA, whereas GC remission rates (BVAS of 0 plus PSL of ≤10 mg/day) were 40 % for MPA and 39 % for GPA. In patients with generalized or severe type diseases, remission rates at month 6 were 84 % for MPA and 83 % for GPA. Time to remission was similar between MPA and GPA patients, and almost all enrolled patients achieved remission at least once during the observation period. These results are probably comparable with the results reported in RCTs conducted in Europe [19, 20]. In the CYCAZAREM trial [19], 155 patients with generalized GPA and MPA received oral CY and PSL for at least 3 months, and patients who achieved remission by 6 months were assigned to treatment with azathioprine or oral CY. A total of 144 of 155 patients (93 %) achieved remission with a BVAS of 0 by 6 months. In the CYCLOPS trial [20], where 149 patients with generalized GPA and MPA received intravenous pulses of CY or oral CY for at least 3 months, 131 patients (78.9 %) achieved remission by 9 months.

Fourteen of 78 MPA patients and two of 33 GPA patients died during the 24-month observation period. The mortality rate of 14 % in our cohort appears to be slightly higher than the rates found in the European RCTs. For example, eight deaths in 155 patients (5 %) within 18 months and 14 deaths in 149 patients (9 %) within 18 months were reported in the CYCAZAREM and CYCLOPS trials, respectively [19, 20]. This difference may be partly attributable to the fact that the mean age at the first presentation was higher in our cohort (71 years for MPA and 64 years for GPA) and a higher proportion of patients with EUVAS-defined severe type disease were enrolled in our study (20 %). The latter possibility is supported by the high mortality (35 deaths in 137 patients; 26 %) demonstrated in the MEPEX trial that selectively included severe type MPA and GPA patients. It is intriguing that the MEPEX trial included more than twice as many MPA patients as GPA patients, similar to our study. A recent comparison of MPA patients between Japan and Europe showed no difference in cumulative patient survival [12, 15]. Thus, these results suggest that MPA may have a worse vital prognosis than GPA, at least in part due to the effects of age at onset.

Further, a marked difference between patients with MPA or GPA in our cohort was found in terms of renal failure. During the observation period, 13 ESRDs occurred in MPA patients, whereas no ESRDs were reported in GPA patients, clearly indicating that ESRD-free survival is much worse with MPA. In agreement with this finding, a retrospective analysis of a large-scale AAV patient database demonstrated that MPO-ANCA-positive patients presented with worse renal function and higher levels of proteinuria, probably due to advanced chronic damage with a smoldering clinical course, than PR3-ANCA-positive patients [21]. Hauer et al. [22] reported that the glomerular filtration rate (GFR) at entry and predominantly chronic renal lesions were potent predictors of the GFR at 18 months in ANCA-associated glomerulonephritis. In the present study, MPA patients had a lower average estimated GFR than GPA patients (38 versus 52 ml/minute/1.73 m^2^). These data would explain the significant difference in ESRD-free survival between MPA and GPA patients in our cohort.

The relapse rate of 23 % in our cohort was similar to the rate reported recently in China (32.7 %) [23] but was lower than the rates reported previously in western countries (45–76 % at 6 months and 38 % at 1 year) [24, 25]. The higher relapse-free survival rates in our cohort and the Chinese cohort may be related to the predominance of MPO-ANCA positivity in Asian patients with AAV, because MPO-ANCA-positive AAV patients relapse less frequently than PR3-ANCA-positive patients [16]. There is also a possibility that relatively slow tapering of GC contributed to a higher relapse-free survival in this study. Although the British clinical guideline recommends rapid tapering of PSL to 15 mg/day after 3 months of treatment [8], more than half of our patients were still taking ≥15 mg/day PSL at month 3. However, it is highly controversial whether fast tapering of GC is associated with increasing risk of relapse in AAV patients. While some studies supported this notion [26, 27], it has recently been demonstrated that GC therapy beyond 6 months is associated with a greater risk of infections, but not with a decreased risk of relapse [24]. The incidence of SIs in the present study was notably higher than those in previous observational studies from European countries [24, 28]. The high incidence of SIs may relate to this slow tapering of GC. On the contrary, VDI scores were comparable with previous RCTs [29].

Our study has several limitations. First, institutional bias should be considered, because this study was performed in university and referral hospitals in Japan. Because the participating institutions enrolled all patients with newly diagnosed AAV, the selection bias in each institute was low. Second, we could not perform multistratified or multivariate analysis for outcomes because of the small sample size. In addition, we could not perform sufficient comparisons between MPO-ANCA-positive and PR3-ANCA-positive patient groups, because there are few numbers of PR3-ANCA-positive patients in this cohort and the observational period was short. This problem will be solved in the future by combination with another large-scale cohort study of Japanese patients with AAV that is currently ongoing. Third, the 2-year observation period could be too short to evaluate the relapse rates of the disease. Fourth, our effectiveness data may be affected by indication bias because treatments were determined at the discretion of the attending physicians. More sophisticated statistical methods should be employed to overcome this bias with a larger number of patients.

## Conclusions

The majority of Japanese patients with MPA and GPA received treatment with high-dose GC and limited CY use, and showed high remission and relapse-free survival rates but low GC remission rates in clinical practice. Further studies are needed to optimize treatment for Japanese patients with AAV, who exhibit different clinical characteristics than patients in western countries in terms of genetic backgrounds, disease categories, and ANCA serology.

## Abbreviations

AAV: Antineutrophil cytoplasmic antibody-associated vasculitis
ANCA: Antineutrophil cytoplasmic antibody
BVAS: Birmingham Vasculitis Activity Score
CY: Cyclophosphamide
EGPA: Eosinophilic granulomatosis with polyangiitis
EMEA: European Medicines Agency
ESRD: end-stage renal disease
EULAR: European League Against Rheumatism
EUVAS: European Vasculitis Study Group
GC: Glucocorticoids
GFR: Glomerular filtration rate
GPA: Granulomatosis with polyangiitis
ILD: Interstitial lung disease
MPA: Microscopic polyangiitis
MPO: Myeloperoxidase
PR3: Peroxidase-3
PSL: Prednisolone
RCT: Randomized controlled trial
RemIT-JAV: Remission Induction Therapy in Japanese patients with ANCA-associated Vasculitides
RLV: renal-limited antineutrophil cytoplasmic antibody-associated vasculitis
SI: Serious infection
VDI: Vascular Damage Index
